# Comparison of gestational diabetes mellitus and pre-eclampsia in women with high hemoglobin in the first trimester of pregnancy: A longitudinal study

**DOI:** 10.12669/pjms.294.4012

**Published:** 2013

**Authors:** Ferdous Mehrabian, Seyyed Mohammad Hosseini

**Affiliations:** 1 Ferdous Mehrabian, MD, Associate Professor of Obstetrics & Gynecology, Obstetrics & Gynecology Department, Faculty of Medicine, Isfahan University of Medical Sciences, Isfahan, Iran.; 2 Seyyed Mohammad Hosseini, MD, Physician, Obstetrics & Gynecology Department, Faculty of Medicine, Isfahan University of Medical Sciences, Isfahan, Iran.

**Keywords:** Antenatal visit, Hemoglobin, Preeclampsia, Gestational diabetes mellitus

## Abstract

***Objective:*** To determine the association between high hemoglobin with gestational diabetes mellitus (GDM) and preeclampsia in pregnant women in the first trimester.

***Methods: ***This cohort study was conducted among 973 pregnant women who started their antenatal booking in the first trimester (first 14 weeks of gestation). Women with first-visit high Hb levels (> 12.5 g/L) on first visit of the pregnancy period were selected as the study group and were compared with those who had normal Hb value (< 12.5 g/L) as controls. Adverse pregnancy outcomes including preeclampsia and GDM were compared between the two groups.

***Results: ***Complete obstetric records of 448 women with high Hb levels and 486 women with normal Hb levels were studied. The follow up showed that the women with high Hb levels had significantly higher rates of preeclampsia and GDM than those with normal Hb levels; the risks were 5.4 (95% cl; 2.8 to 10.5) and 3.7 (95%cl; 2.2 to 6.4), respectively.

***Conclusion:*** This study found that high Hb in the first trimester is associated with higher risk of subsequent preeclampsia and gestational diabetes mellitus (GDM).

## INTRODUCTION

Toxemia of pregnancy (pre-eclampsia) is a serious disorder that may occur from the 20^th^ weeks of gestation up to seven days after delivery. It can cause vasoconstriction, and may result in increase in blood pressure and lowering the blood flow. Reduction in uterine blood flow can cause serious problem such as reduced fetal growth, decreased amniotic fluid and placental abruption. In addition, If the preterm delivery would be necessary, the fetus may be born prematurely.^1^^,^^2^ Despite extensive research, the main cause of this disease is still not perfectly known. Measurement of hemoglobin (Hb) is a standard test for evaluation of physical status and anemia among pregnant women in their first visits. High level of hemoglobin is the evidence of adequate nutrition and health. However, high level of hemoglobin is dangerous and this will be always monitored by caregivers.

Conflicting results have been obtained in pregnant women with high hemoglobin levels regarding preeclampsia, gestational diabetes mellitus(GDM), preterm labor, short for gestational age(SGA) and stillbirth.^3^^-^^9^^. ^Many studies have shown that Hb in circulation after oxidation could be the cause of methemoglobin-derived heme deposition on the vascular endothelium. This heme, in turn, can increase vascular damage and developing arthritis in the endothelium^10^^,^^11^ blood vessels autoerotic as rampant in umbilical level of pregnancies with preeclampsia.^12^ In addition, heme as a catalyst acts to oxidize LDL (low-density lipoprotein) and induce toxicity on the vascular walls.^13^^,^^14^ Early diagnosis of preeclampsia can improve the prenatal care for pregnant women during pregnancy and also result in satisfactory outcome of the pregnancy.

Few studies have been conducted on the relationship between high levels of maternal hemoglobin with preeclampsia and gestational diabetes in the first trimester. Studies conducted by Vega et al. in relation to maternal hemoglobin levels revealed a significant relationship between hemoglobin levels and developmental abnormalities.^15^ Maternal hemoglobin levels can indicate the status of the embryo. Its increase by more than 146 milligrams per deciliter can increase the incidence of stillbirths.^16^ High levels of maternal hemoglobin in the first trimester and the incidence of preeclampsia, gestational diabetes and early detection of these disorders does affect the preconception care in this group of mothers. We planned this comparative study to find out the relationship of gestational diabetes and preeclampsia in pregnant women with high and normal hemoglobin in the first trimester.

## METHODS

This prospective cohort study was conducted in 2011-2012. Pregnant women who were referred for their routine examination, before the fourteenth week of pregnancy in the first trimester, to the clinics affiliated of the Isfahan University of Medical Sciences were included in the study.

***Inclusion criteria:***

1. No previous history of gestational diabetes
2. No previous history of pre-eclampsia
3. No history of molar fetal hydrops multiple pregnancy
4. No history of chronic hypertension
5. Non-smoking
6. Not suffering from Thallasemia
7. Completion of the informed consent to participate in the study

***Exclusion criteria:***

1. The patient's desire to withdraw from the study in each stage
2. High hemoglobin greater than 11 g/l due to other diseases (chronic hypertension, kidney disease, diabetes mellitus, etc.)
3. Neonatal malformations in the ultrasound of the pregnancy

Sampling was based on non-random convenient sampling. Those who met the inclusion criteria were entered into the study with the justification of the study purposes after obtaining their informed consent. Then, their demographic characteristics and gestational age (The first day of the last menstrual period) were recorded in the first trimester before the 14^th^ weeks. All subjects were tested and divided into two groups based on hemoglobin levels. After calculating the cut off point of hemoglobin (Hb ≥ 12.5 g/l), patients with Hb = 12.5 g/l were studied as a group and those with normal hemoglobin (lower than 12.5 g/l) as the control group. The patients were followed up from gestational diabetes and preeclampsia until delivery. Ultimately, the number and frequency of the patients with gestational diabetes and preeclampsia were documented in each group.

Diagnosis of preeclampsia was based on systolic blood pressure of the patient equal to or greater than 140 mm Hg and diastolic blood pressure equal to or greater than 90 mm Hg in the two measurements of six hours plus 300 mg proteinuria in 24 hours equal to or greater than +1 in two urine samples.

Diagnosis of gestational diabetes was based on positive gestational diabetes screening test of the patient. Oral glucose tolerance test (OGTT) was performed initially regardless of the interval between the test and last meal, 50 g of glucose was given to the patient and blood glucose levels were measured one hour later. If the obtained value was equal to or greater than 140 milligrams per deciliter, then the three-step test was performed. Patients were asked to come to the laboratory after an overnight fasting; they were also asked to follow their usual diet without restricting its carbohydrate content during the three days without restrictions. Initially, a fasting blood sample was taken, then the blood samples were taken with intervals of one, two and three hours after taking 100 grams of glucose. If two of the values out of four ​​obtained values were higher than normal, the diagnosis of gestational diabetes was made.

## RESULTS

Nine hundred seventy three (973) pregnant women were included in this study. Twenty nine participants were excluded due to incomplete information. Finally, data on 944 patients in the two groups were included in the final analysis. The first group included 458 pregnant women with hemoglobin levels greater than 12.5 g/l. The second group included 486 pregnant women with hemoglobin levels less than 12.5 g/l (normal). The mean age of the participants was 27.6 ± 5.6 years with a median of 28 years. The youngest patient was 18 years old and the oldest one was 42 years old. The frequency of studied subjects is given in Table-I Based on the T-test results, there were no significant differences between the two groups regarding the distribution of individuals in age groups (p-value = 0.3).

**Table-I:** Frequency distribution of studied pregnant women by age groups

*Age group (years)*	*All subjects*		*Normal Hb*		*High Hb*		*P-value*
	*No.*	*%*	*No.*	*%*	*No.*	*%*	
> 20	61	6.5	34	7	27	5.9	0.3
20-34	752	79.7	392	80.7	360	78.6	
≤ 35	131	13.9	60	12.3	71	15.5	
Total	944		486		458		

**Table-II:** Relative frequency of preeclampsia among pregnant women in the two groups studied

		*High Hb group*		*Normal Hb group*		*P-value*
		*No.*	*%*	*No.*	*%*	
Preeclampsia	+	51	11.1	10	2.1	< 0.0001
	-	407	88.9	476	97.9	
Total		458		486		

**Table-III:** Relative frequency of gestational diabetes among pregnant women in the two groups studied

		*High Hb group*		*Normal Hb group*		*P-value*
		*No.*	*%*	*No.*	*%*	
Gestational diabetes	+	56	12.2	16	3.3	< 0.0001
	-	402	87.8	470	96.7	
Total		458		486		


**Table-IV:** Relative risks and 95% confidence intervals for preeclampsia and gestational diabetes in the studied subjects

	No.	High Hb N = 486		Normal Hb N = 458		RR (95% CI)	
Preeclampsia	61	51	11.1%	10	2.1%	5.4	2.8-10.5
Gestational diabetes	72	56	12.2%	16	3.3%	3.7	2.2-6.4

In the study of relative frequency, it was identified that there was preeclampsia in 61 patients (6.5%) out of the total patients and 51 subjects among them, were in the high-hemoglobin group and 10 patients were in the normal hemoglobin group. According to the results of the chi-square test presented in Table-II, there was a significant difference between the two groups regarding the rate of preeclampsia (p-value < 0.0001).

In the investigation of relative frequency of gestational diabetes, 72 patients (7.6% of the total patients), were diagnosed as gestational diabetes, while 56 of them were in high hemoglobin group. Sixteen patients were in the group with normal hemoglobin. According to the results of the chi-square test, which are presented in Table-III, there was a significant difference between the two groups. The rate of gestational diabetes (p-value < 0.0001) is shown in Table-IV, in addition to the rate of relative risk and 95% confidence intervals for preeclampsia and gestational diabetes in studied subjects. The relative risk for preeclampsia in pregnant women with high hemoglobin (2.8-10.5) was 4.5 times more than pregnant women who had normal hemoglobin. The frequency of women with GDM in pregnant women with high hemoglobin was significantly greater than in pregnant women with normal hemoglobin.

## DISCUSSION

This study showed that the risk for gestational diabetes in pregnant women with high hemoglobin level was 3.7 times more than the obtained results with pregnant women with normal hemoglobin. Phaloprakarn and colleagues,^14^ conducted a study on 920 pregnant women in the two groups with hemoglobin above 12.5 and below 12.5 g/l. The incidence of preeclampsia in pregnant women with high hemoglobin levels was 11.5 percent vs. 2.9 percent in pregnant women with normal hemoglobin levels. This difference was reported statistically significant. The relative risk in high hemoglobin group was 4.3 times more than the normal hemoglobin group. These results were similar to findings in our study in which the rate of preeclampsia was 11.1 in high hemoglobin group versus 2.1 in the group who had normal hemoglobin levels. The obtained relative risk in the present study was 5.4 (2.8-10.5). In the study of Phaloprakarn and colleagues,^14^ gestational diabetes in high-hemoglobin group compared with normal hemoglobin group achieved more (11.7 vs. 2.1, respectively) with relative risk of (2.1-6.9), which was 2.8 times higher than the high hemoglobin group compared with normal hemoglobin group. These results were also consistent with the results of the present study. The incidence of gestational diabetes in pregnant women in high hemoglobin group compared with normal hemoglobin group, obtained (12.2 percent vs. 3.3 percent, respectively) with the relative risk of 3.7 (2.2-6.4).

Murphy and colleagues,^4^ studied 44,316 pregnant women, which showed a high incidence of preeclampsia in women who had Hb ≥ 133 g/l in their first prenatal visit compared with women who had Hb = 104-132 g/l. The results were consistent with the results of the present study. Despite differences in hemoglobin levels as the level of significance, the impacts of high hemoglobin levels were demonstrated on increasing the rate of preeclampsia in pregnant women. In another study carried out by Ren and colleagues (2007), about the relationship between low hemoglobin in 88,149 pregnant mothers who were under the care of general pregnancy in China observed an increased incidence of LBW, SGA and preterm labor in the first trimester.^17^ Another study conducted in 2002 by Trence and colleagues in 730 pregnant mothers found that maternal hemoglobin above 13 had a higher risk of developing GDM.^18^ Tarim et al also looked at the incidence of GDM and high maternal hemoglobin with high ferritin. In this prospective study, 253 non-diabetic pregnant women who had hemoglobin and ferritin levels higher than 100 in the first trimester were investigated. In this review, pregnant women with serum hemoglobin of 122 had significant risk of developing gestational diabetes.^6^

 In another study, which was conducted in 1998 by Ming Zhou and colleagues, 900 women from 23 districts were recruited randomly. This study looked at the relationship between the measured hemoglobin in different times. Mothers who had lower hemoglobin than 110 were faced with a greater risk of LBW and preterm labor.^19^ In this study, the maternal hemoglobin concentration in women with GDM was higher compared to non GDM women. This difference was statistically significant. Similar results were obtained particularly high levels of GDM in pregnant women with high hemoglobin compared with women with normal hemoglobin in various other studies.^5^^,^^6^^,^^20^^,^^21^ In Gungor and colleagues’ study^22^ in 2007, unlike the present study, there was no significant relationship between the hemoglobin in the first trimester with gestational diabetes mellitus (p-value > 0.05). The difference in determining gestational age for measuring hemoglobin in Gungor study comparing with the present study could be due to differences in the studies. In this study, hemoglobin in the first trimester was considered, but in the study of Gungor, hemoglobin has been investigated in the weeks of 28-30 of gestation. Probably, it did not identify the real difference in hemoglobin levels between the groups due to the effect of iron supplementation in the second half of pregnancy.

## CONCLUSION

High hemoglobin levels greater than 12.5g/dl in pregnant women was associated with an increase in the risk of gestational diabetes and preeclampsia. Hence it is recommended that women with high hemoglobin in their first trimester should be considered at greater risk of developing gestational diabetes mellitus and pre-eclampsia. Early detection and prevention will reduce the undesirable effects.
